# Protocol for the STAR (Sheffield Treatments for ADHD) project: an internal pilot study assessing the feasibility of the Trials within Cohorts (TwiCs) design to test the effectiveness of interventions for children with ADHD

**DOI:** 10.1186/s40814-018-0250-3

**Published:** 2018-03-02

**Authors:** Philippa Fibert, Clare Relton, Tessa Peasgood, David Daley

**Affiliations:** 1 School of Health and Related Research, University of Sheffield, Regent Court, 30 Regent Street, Sheffield, S1 4DA UK; 2 Institute of Mental Health, Jubilee Campus, University of Nottingham, Wollaton Road, Nottingham, NG8 1BB UK

**Keywords:** ADHD, Feasibility, TwiCs, Nutrition, Homoeopathy

## Abstract

**Background:**

Attention deficit hyperactivity disorder (ADHD) is a common and growing problem and a leading cause of child referrals to Child and Adult Mental Health Services (CAMHS). It is a drain on resources across nationally funded support agencies and associated with negative outcomes such as early criminality, school disruption and antisocial behaviour. Mainstream interventions (pharmacological and behavioural) demonstrate effectiveness whilst implemented, but are costly, often have unwanted side effects and do not appear to be affecting long-term outcomes.

Development of a robust evidence base for the effectiveness of current and novel interventions and their impact over the long term is required. The aim of the Sheffield Treatments for ADHD Research (STAR) project is to facilitate a rigorous evidence base in order to provide information about the comparative (cost) effectiveness and acceptability of multiple interventions to key stakeholders.

**Methods:**

The Trials within Cohorts (TwiCs) design was used to build a cohort of children with a diagnosis of ADHD and conduct a three-armed pilot trial of the clinical and cost effectiveness of two novel interventions: (a) treatment by nutritional therapists and (b) treatment by homoeopaths, compared to (c) treatment as usual.

Participants are recruited to the STAR long-term observational cohort, and their outcomes of interest (ADHD symptoms, health-related quality of life, school disruption, resource use and criminality) are measured every 6 months by carers and (blinded) teachers. Two promising interventions were identified for the first randomised controlled trial embedded in the cohort. A random selection of eligible participants is offered treatments (a) and (b). The outcomes of those offered treatment are compared to those not offered treatment using intention to treat (ITT) analysis.

The feasibility of recruiting to the cohort and the trial, delivering the interventions, the effectiveness of the interventions and the appropriateness, sensitivity and collectability of outcomes is trialled.

**Discussion:**

The results of this trial will provide information on the feasibility of the TwiCs design to facilitate multiple trials of potential interventions for children with ADHD, and the acceptability, clinical and cost effectiveness of two potential interventions for ADHD to ADHD stakeholders including service providers. Future stages of the STAR project will test other treatments informed by the results in stage 1.

**Trial registration:**

ISRCTN number 17723526. 10.1186/ISRCTN17723526. Date assigned 27/4/15.

## Background

ADHD is a common and growing problem and a leading cause of child referrals to Child and Adult Mental Health Services (CAMHS). It accounts for a sizeable amount of resource use, is a drain on resources across nationally funded support agencies [1], and presents a major, often unmet challenge to services [2]. There is a need to improve outcomes, where the condition is associated with negative outcomes such as early criminality, school disruption and antisocial behaviour. Mainstream interventions (pharmacological and behavioural) demonstrate effectiveness whilst implemented, but are costly, often have unwanted side effects and do not appear to be affecting long-term outcomes.

ADHD is associated with a range of negative behaviours such as difficulties regulating emotions, particularly anger [3], involvement with violent criminality and at a young age [4], school disruption, exclusion and low attainment [5, 6]. Children often have few friends [7], and their experience of school can be one of academic failure, rejection by peers and low self-esteem [8]. Information is required about cost-effective interventions which can achieve positive changes over the long term.

Current interventions may only be effective whilst implemented [9, 10] over the short term [11–13] and compliance with medication (the main intervention) can be poor [14, 15]. Furthermore, up to 25% of children may not respond to stimulant medication [16–18] which is associated with adverse events [19] particularly those with concomitant ADHD and autism spectrum conditions (ASCs) [20–22].

Whilst we have information about the efficacy of mainstream interventions, it is unclear whether this translates into real-world effectiveness, acceptability and improvement in long-term outcomes. Pragmatic trials will be helpful for a variety of reasons [23]. Measurement of the acceptability and (cost) effectiveness of interventions over the long term can provide important information for stakeholders. Heterogeneity is a feature of ADHD expression and children often have a wide range of other diagnoses such as ASCs [24], conduct disorders [25], sleep disturbance [26], gut dysbiosis and tics [27]. Information is required about whether efficacious interventions for children with single ADHD diagnoses are effective in populations with additional co-morbidities. Finally, children with ADHD and their families can be hard to engage and treat [28]; therefore, the acceptability of interventions is an important consideration.

The Trials within Cohorts (TwiCs) design (also known as the cohort multiple randomised controlled trial (cmRCT) design) has been developed to help address some of the limitations associated with existing RCT designs [29]: for example, the tendency for potential treatments to be trialled one at a time, in different populations by different research teams, yielding trials with heterogeneous trial populations between trials and often short-term and heterogeneous outcomes.

The TwiCs design recruits a large, long-term observational cohort of people with the condition of interest and regularly measures their outcomes of interest. For each trial embedded within this cohort, a proportion meeting trial inclusion and exclusion criteria are offered interventions. Those not randomly selected act as a virtual treatment as usual (TAU) control group.

The STAR project aims to facilitate a rigorous evidence base in order to provide information about the comparative (cost) effectiveness and acceptability of multiple interventions to key stakeholders about interventions which might address treatment gaps. This pilot study addresses the research question: Is the TwiCs design feasible to assess the effectiveness of treatments for ADHD? For the study, two interventions with preliminary indications of effectiveness, considered complementary and alternative medicine (CAM) [30], have been selected. Parents of children with ADHD are trying these interventions, paying for them out of pocket.

CAM use for ADHD has been found to range from 12% [31] to 71% [32]. Its use is increasing, especially for conditions without effective recommended treatments, where medical treatment is associated with serious adverse effects [33] and for children with chronic conditions [34–36]. Patients are using CAM despite physician’s concerns, and in addition to medication, therefore, development of a robust evidence base is required. The CAM interventions being used by parents of children with ADHD include acupuncture, aromatherapy, chiropractic, elimination diets, fatty acid supplementation, herbal medicine, homoeopathy, massage, speech, physical and occupational therapy, and vitamins [31, 32, 36, 37].

For this first RCT, we identified two interventions showing indications of effectiveness and being used by parents of children with ADHD. Since they are being tested as experienced in real-life clinical practice as befitting a pragmatic trial, we named them treatment by a homoeopath and treatment by a nutritional therapist.

The published evidence for homoeopathic treatment for ADHD consists of seven trials: two RCTs tested the efficacy of specific homoeopathic formulas [38, 39]; four RCTs tested the efficacy of individualised homoeopathic medicines [40–43]; and one within subjects trial of children with ASCs measuring ADHD symptoms tested the effectiveness of individualised homoeopathic treatment [44]. All studies but one [42] of individualised homoeopathy found significant improvements, but effect size varied, and quality is variable.

Although a healthy diet is recommended by NICE [2], no trials assessing the effectiveness or acceptability of this have yet been conducted. There are many studies exploring the efficacy of individual nutrients, multiple nutrients, and special diets for ADHD. Systematic reviews concur that polyunsaturated fatty acids have small effects on ADHD symptoms [45, 46] and that restricted elimination diets, artificial food colour elimination and dietary supplements may be efficacious, but more studies are required before firm conclusions can be reached. Improvements may be optimised by both eliminating aggravating factors and boosting nutritional deficits in an individualised way, since sensitivities are found in some but not all children with ADHD. Treatment by nutritional therapists provides advice about, and support implementing, individually tailored exclusion, supplementation, and healthy eating.

### Aims and objectives

The aim of the STAR project is to provide a facility for efficiently testing multiple interventions for children with ADHD.

Stage 1 of the STAR project will test the feasibility of the Trials within Cohorts (TwiCs) design and conduct a three-armed pilot trial of the clinical and cost effectiveness of two interventions: (a) the offer of a course of treatment by homoeopaths and (b) the offer of a course of treatment by a nutritional therapist, compared to (c) treatment as usual.

Stage 1 will test the feasibility of the TwiCs design by conducting a small-scale test of the methods and procedures and assess the acceptability, deliverability, clinical and cost effectiveness of treatment by homoeopaths and nutritional therapists for children with ADHD.

The objectives of stage 1 are to assess the feasibility of recruiting to time and target a cohort of children with a diagnosis of ADHD, their carers and teachers; the feasibility and acceptability of the study design; the feasibility, deliverability, safety and acceptability of the interventions; the suitability, acceptability and deliverability of the outcome measures; and to inform sample size calculation for a full trial (stage 2).

## Methods

### Recruitment

At least 140 children aged 5–18 with a diagnosis of ADHD and any accompanying co-morbidities are recruited to an observational cohort and their consent to participate in the cohort is sought by completion of a questionnaire with embedded consent to be contacted again. Their outcomes of interest are measured at recruitment (baseline) and every 6 months, via these questionnaires, which are completed by carers and blinded teachers. Carers of children with ADHD were involved in the selection of items for, design and pilot testing of the questionnaire, advised about recruitment and sat on the steering committee.

### Randomisation

After recruitment to the STAR cohort, a three-armed internal pilot trial is subsequently conducted whereby random selections of eligible children are offered either adjunctive treatment by homoeopaths (arm 1) or nutritional therapists (arm 2). Randomisation is computer generated by an independent statistician from the University of Sheffield and held in the locked drawer of a different statistician from the University of Sheffield. Stratifying factors are age (primary 5–11/secondary 12–18), medication status (yes/no) and ADHD severity (Conners global ADHD *T* score 80+/under 80) [47]. Participants fulfilling inclusion and exclusion criteria who consent to be contacted again are identified on recruitment to the cohort. Stratifying factors are extracted and emailed to the statistician holding the randomisation list, who randomly assigns them to one of the three groups and gives them a randomly generated three-digit code by which they are subsequently identified.

Carer and child’s consent to try treatments is sought at this stage. They are sent a letter of invitation for 1 year of treatment, which includes a statement that participants can withdraw at any time; a brief description of the treatment; requests the carer to ask their child if they are happy to participate; a consent form to treatment to be signed by carer and child and returned in a pre-paid envelope; and an email address and telephone number as an alternate means of contact. The PI then rings the participants up and asks if they have any questions; if they wish to participate (if their form has not yet been returned); and if they both consent, to their details being passed on to the therapist. In the absence of a returned ‘consent to treatment’ form, carer and child’s wish to participate on the telephone is taken as verbal consent. Consent forms are then signed at the first appointment with the therapist, who further explains what treatment will constitute, as befits usual practice. Appropriate marks by young children are taken as signatures. Those children not selected act as a treatment as usual control (arm 3).

### Sample size

Sample size for the pilot trial is calculated to recruit at least 30 participants in each arm in order to estimate the pooled standard deviation of the outcome with a reasonable degree of precision for the full trial [48, 49]. We have estimated that attrition will be 20% based on a preliminary feasibility-controlled case series [50] and that a further 20% joining the STAR cohort will either not meet the first trial inclusion criteria or not accept the offer of treatment.

Sample size for the full trial will be based upon results obtained in the pilot. The calculation will be based upon the obtained effect size (SMD) for the primary outcome, significance (alpha error) of 0.05 and power of 80% and adjusted according to the levels of attrition observed. If an effect size of .4 is obtained in the pilot, then the outcomes of approximately 100 participants per group will be required. If estimated attrition of 40% is correct, then 166 participants per group will need to be recruited to the cohort.

Participants are recruited from registered charities, schools, word of mouth and support groups. The majority of participants are recruited from the city of Sheffield; however, participants are also recruited nationally and the feasibility of on-line treatments assessed.

### Inclusion/exclusion criteria

Inclusion criteria for the STAR cohort are children aged 5–18 (inclusive) with a carer-reported diagnosis of ADHD (date, venue and diagnosing physician) and a carer-reported Conners Global Index (CGI) *T* score of at least 55 (denoting mild atypicality (Table 1). Inclusion criteria for the trial are a carer-reported CGI score of at least 65 (indicating a significant problem) (Table 1).

**Table 1:** Interpretive guideline for Conners’ *T* scores and percentiles

*T* score	Percentile	Guideline
70+	98+	Markedly atypical (significant problem)
66–70	95–98	Moderately atypical (significant problem)
61–65	86–94	Mildly atypical (possible problem)
56–60	74–85	Slightly atypical (borderline)
< 30–55	< 2–73	Average (typical: should not raise concern)

Exclusion criteria to both cohort and trial are children below the age of 5 or adults over 18; children with terminal, severe, life-limiting conditions; and families where English is not written or spoken (as the project does not have funds to provide a translation service). Exclusion criteria to the trial are children currently receiving treatment by a homoeopath or a nutritional therapist and children with CGI *T* scores below 65.

### Outcome measurement

Carers of children with ADHD are asked to complete carer questionnaires (CQs) sent to them by post or completed on-line (www.starsheffield.com).The CQ includes the 10-item Conners’ Global Index (CGI) [47]; the 18-item Swanson, Nolan and Pelham Teacher and Parent Rating Scale (SNAP) [51]; the 9-item Child Health Utility health-related quality of life (CHU-9) measure [52]; and questions about resource use. The last page asks for consent from carers and children to be contacted again, for their school to be contacted, personal and school contact details.

Where consent is given, schools are sent a teacher’s questionnaire (TQ) and an information letter. They are not informed about treatment allocation. The TQ includes the CGI, SNAP, and questions about school-specific resource use, attendance, disruption and exclusion. Schools which do not want to participate are not contacted again.

Control group participants are not informed that they have not been selected for a treatment but continue to be sent questionnaires at the same time intervals as treatment groups. CQs and TQs are sent out every 6 months to all cohort participants. Those refusing the offer of treatment are still asked to complete outcome measures as usual every 6 months and considered members of the offer group.

### The interventions

Homoeopaths are registered with one of the four main bodies representing homoeopaths in the UK. Nutritional therapists are registered with the British Association of Applied Nutrition and Nutritional Therapy and are members of the Complementary and Natural Healthcare Council. All therapists are CRB checked, complete an on-line NSPCC child protection course (https://www.nspcc.org.uk/what-you-can-do/get-expert-training), attend a 1-day training course on risk and receive guidelines in management of ADHD and serious adverse events.

Both interventions are offered to participants for a maximum of 1 year consisting of a maximum of eight appointments. Consultations take place at Complementary Medicine Centres, participant’s homes or on-line. First consultations last for up to 1½ h and follow-ups last up to 40 min approximately every 6 weeks for up to 1 year resulting in a total of up to seven contact hours. Homoeopaths conduct fact-finding consultations on the basis of which they prescribe homoeopathic medicines, guided by underlying basic homoeopathic principles [53]. Treating ‘like with like’ is the core principle: that substances causing certain symptoms in healthy persons may cure those same symptoms in those who are ill. Nutritional therapists take an initial health history and discuss and agree with participants an appropriate range of options such as elimination diets, healthy food and menu options, reducing aggravators such as food colourings or sugar; improving fluids intake, lifestyle issues such as sleep; specific dietary intervention for symptoms; and supplementation.

At each consultation, therapists ask participants and/or their carer (dependent on age) to complete Measure Your Own Medical Outcome Profile (MYMOP) [54]. This patient generated outcome measure consists of 4 self-chosen items measured using a Likert-type scale of 0 (as good as it could be) to 6 (as bad as it could be): two symptoms considered most bothersome, an activity ADHD limits participation in, and well-being. Diminution of scores represents improvement. This personalised measure allows participants to select their key concerns and document how they change over time. It is a validated outcome measure considered appropriate to capture non-specific effects.

Therapists also ask about any adverse events experienced by participants which are recorded according to the Common Terminology Criteria for Adverse Events guidelines [55]. Therapists are instructed to report adverse events of level 3 or more to the PI within 24 h, who refers them immediately to the study Data Management and Ethics Committee for appropriate action.

Information regarding adverse events for children with ADHD in response to homoeopathic treatment suggests that there may be occasional mild transient increases in symptoms, which are however usually associated with overall feelings of well-being. Information in response to nutritional therapy suggests that there may be occasional minor side effects from supplementation.

### Outcomes

#### Patient-centred outcomes

The primary outcome measured is ADHD symptomatology measured using CGI, consisting of a 10-item total score and two sub-scores measuring restlessness/impulsivity (7 items) and emotional lability (3 items). The outcome is reported by carers (unblinded) and teachers (blinded), and each intervention is compared to usual care. Secondary outcomes are carer proxy health-related quality of life (CHU-9) and sleep. Resource use is collected by asking about medication use; further ADHD interventions being used; hospital, GP, social worker and police attendances; levels of absenteeism, exclusion and criminality; and teaching assistant and other help in school.

Resource use data is collected to enable an economic evaluation alongside a clinical trial. Evaluation will include health-related quality of life assessment, with utility measures based on the CHU-9D, the cost of consultations, medications, remedies, supplements and room hire, to enable QALY and ICER calculation. The time horizon is 1 year, during which time resource use will be collected from therapists, who complete therapist forms at each consultation documenting the time spent, the venue, medications or supplements given.

#### Feasibility outcomes (Table 2)

Key feasibility criteria are the ability to recruit a cohort and to recruit to the interventions within the stated time frame of 2 years, by measuring recruitment rates and time taken. Numbers recruited in 2 years will be extrapolated to estimate the time needed to recruit to a full trial, and longer than 4 years will not be considered feasible. The feasibility of recruiting a representative cohort will document ADHD severity, number and type of co-morbidities, medications taken, resource usage, levels of exclusion, and criminality, and recruiting from nationally funded ADHD facilities such as special schools, secure units, NHS facilities and support services.

**Table 2:** Feasibility criteria

Criteria	Measurement	Criteria for continuation to a full trial
Recruitment to cohort rates	Number recruited in 2 years	% recruited/sample size estimation. Minimum acceptable to proceed to a full-scale trial will consider the number of years needed to recruit the required sample size. Numbers recruited in 2 years will be extrapolated to determine how long a full trial would need to be. The required sample size will be divided by the percentage recruited in 2 years. Duration of the full trial of more than 4 years will not be considered feasible.
Recruitment to treatment rates	Percentage accepting offer	At least 30%. The percentage of participants refusing treatment will be considered to determine acceptable levels of refusal. Too many treatment refusers may lead to a high chance of a type II error and inadequate information to estimate critical parameters for a full trial with reasonable precision.
Treatment effects (statistical significance)	Standard mean difference (SMD) CGI	Mean = < .3. Since neither intervention has been tested in this form previously, estimation of the effect size cannot consider previous estimates. Since we need to know that therapies show some evidence of being helpful, a SMD of 0.3 in those implementing the therapies will be considered sufficient to proceed to full trial.
Treatment effects (clinical significance)	CGI *T* score	5 percentiles. A *T* score change of 5 percentiles is considered clinically significant, since the child has moved from one level of severity to another according to Conners (2009) [47].
Attrition: cohort	Number of PQs returned at 6 months	At least 30%
Attrition: consultations	Number of consultations attended	Adjustment of intervention provision
Acceptability of TQ and CQ	Number of TQs and CQs completed at baseline and 6 months; number of email/telephone/ paper responses	Adjustment of measure, collection methods and trial parameters. Questions reworded or removed dependent on discussion with carers and teachers, and optimum means of delivering questionnaires explored
Adverse events	Clinicians records	No severe adverse events as defined by the Common Terminology Criteria for Adverse Events guidelines
Appropriate outcome measurement	Number of missing items	Adjustment of measure
Recruitment of therapists	Number recruited fulfilling criteria	At least two for each therapy
Statistical analysis	ANCOVA	Meets assumptions

Whether suitable homoeopaths and nutritional therapists can be recruited will be assessed, with recruitment of two therapists for each intervention considered the minimum required. The uptake of treatment will measure the number of appointments kept and missed; the taking of homoeopathic medicines; and the implementation of advice, diets, and the taking of supplements. At least 30% will need to have accessed treatment, and at least 70% of those to have attended at least three consultations, to continue to the full trial. It is anticipated that there may be high dropout rate and chaotic uptake. The percentage of participants refusing treatment will be considered to determine acceptable levels of refusal since too many refusers may lead to high chance of a type II error and inadequate information to estimate critical parameters for a full trial with reasonable precision.

Adverse events will be documented to measure the safety of the interventions using the Common Terminology Criteria for Adverse Events (CTCAE, 2010). Any severe adverse events as a result of treatment will mean the intervention will not continue to full trial.

The acceptability of the outcome measurements will assess rates of completion by parents and teachers at each time point in the study. Collection methods will be adjusted if necessary. Their suitability will also be assessed by ascertaining whether information can be accurately and usefully collected about ADHD symptoms, health-related quality of life, criminality, school exclusion, attendance, extra help, professional resource use, medication and other intervention use and adjusted as necessary. Whether CGI and SNAP are appropriate and sensitive enough to measure therapist- and participant-perceived change will be assessed and measures adjusted accordingly. The measure will be adjusted if any systematically missing items are identified. The suitability and appropriateness of statistical analyses to interpret results will also be assessed.

#### Statistical analysis

Statistical testing is exploratory since the study is not powered to detect statistical differences. It will be used to ensure that a difference can be seen from the interventions to proceed to the full trial, to inform sample size calculation, and to pilot test the statistical analyses selected. Analysis will focus on confidence interval estimation rather than hypothesis testing. IBM SPSS 21 statistical software [56] will be used. All statistical exploratory tests will be two-tailed with significance level (alpha) set to 5%, and 95% confidence intervals presented. Outcomes will be measured by parents and teachers at baseline, 6 months and 1 year. Both interventions tested will be compared with treatment as usual with parent and teacher measurements reported.

Missing data: post hoc tests will be performed to identify any differences between groups regarding whether data is missing completely at random or not at random. 6-month missing data will be imputed using Last Observation Carried Forward or mean substitution (for baseline data) according to tool developer recommendations [47].

‘Intention to Treat’ (ITT) analysis is used whereby participants offered treatment remain within the treatment group regardless of whether they take up the offer of treatment. The method used for statistical analysis of the outcome data will be regression analysis, with outcome change score as the dependent variable and group (homoeopathic treatment or nutritional therapy, analysed separately) as the independent variable. Analysis will control for the effects of gender, ADHD severity (CGI baseline score) and age.

The acceptability of the offer of treatment will be reported by presenting numbers for all patients offered treatment. Statistical tests will be carried out to identify characteristics of those who did and did not accept and receive treatment. Sub group analysis will explore the effect of treatment on those accessing that treatment, those taking/not taking ADHD medication and those with co-occurring autism.

The clinical effect of treatment will be explored by calculating standardised mean differences [57] for those offered and taking up the offer of each treatment to obtain sample size estimates.

## Discussion

The results of this pilot trial will provide preliminary information on the feasibility of the TwiCs design to assess the effectiveness of interventions for children with ADHD to improve (long-term) outcomes. It will also provide preliminary information about the acceptability, clinical and cost-effectiveness of two potential interventions for ADHD. If feasibility criteria are met, then the study will continue to a full trial of these two interventions subject to reviewer’s comments and obtaining further funding.

Future plans are to develop the STAR cohort as a facility where interventions can be rigorously evaluated in a timely and efficient manner. This will entail expanding and ensuring the representativeness of the cohort and offering random samples of eligible participants a variety of mainstream and non-mainstream interventions. Recruitment of a representative cohort will focus on recruiting at-need populations such as teens, particularly those involved in criminality; hard to reach families; those with co-occurring autism; and those with multiple co-morbidities.

Advantages of the TwiCs design are the speedy recruitment of sufficient numbers. No deception is experienced by participants compared with placebo controlled trials. Trial conditions are similar to those patients could expect if consulting therapists in clinical practice and healthcare assessors might expect were they to commission treatments. All types of interventions, for example, complex, therapist-led and/or pharmaceutical treatments can be compared. Since the TwiCs design is designed to test multiple interventions, potential biases are equally distributed across groups.

As a pragmatic trial, a disadvantage is that there is inevitable variation (noise), such as participants trying other interventions, changing medication dosage, or life events. All interventions are equally subject to such noise. A further potential disadvantage is that the offer of treatment, i.e., its acceptability, is measured, since ITT analysis is the primary analysis. The effectiveness of undertaking the treatment (per protocol analysis) is a secondary analysis. Measuring acceptability is an important issue in ADHD research where non-compliance is common. However, assessors may prioritise and require measurement of specific effects within treatments, such as effect due to therapist, supplement, dietary advice, homoeopathic medicine, etc. The real-life effectiveness of the two selected interventions has remained untested until now.

The TwiCs approach can be a cheap way to generate evidence regarding comparative effectiveness and potential to improve long term outcomes and can provide useful information to ADHD stakeholders including service providers. The current approach is inefficient, expensive, slow, difficult to compare, and not focused on improving outcomes for all those with ADHD in the longer term. If feasible, the STAR approach to testing interventions has the potential to contribute to the research canon and to patient outcomes.

## Abbreviations

ADHD: Attention deficit hyperactivity disorder
ASCs: Autism spectrum conditions
CAM: Complementary and alternative medicine
CAMHS: Child and Adult Mental Health Services
CGI: Conners Global Index
CHU-9: Child Health Utility Index (9 items)
cmRCT: Cohort multiple randomised controlled trial
CQ: Carer questionnaire
CTCAE: Common Terminology Criteria for Adverse Events
ScHARR: School of Health and Related Research
SNAP: Swanson, Nolan and Pelham Questionnaire
STAR: Sheffield Treatments for ADHD Research
TAU: Treatment as usual
TQ: Teacher questionnaire
TwiCs: Trials within Cohorts

## References

[1] Scott S, Knapp M, Henderson J, Maughan B (2001). Financial cost of social exclusion: follow up study of antisocial children into adulthood. Br Med J.

[2] NICE (National Institute for Health and Clinical Excellence) Updated Clinical Guideline Number 72. (2013). http://guidance.nice.org.uk/CG72/NICEGuidance/pdf. Accessed 9 Feb 2018.

[3] Edwards G, Barkley RA, Laneri M, Fletcher K, Metevia L (2001). Parent-adolescent conflict in teenagers with ADHD and ODD. J Abnorm Child Psychol.

[4] Young S, Wells J, Gudjonsson G (2011). Predictors of offending among prisoners: the role of attention deficit hyperactivity disorder (ADHD) and substance use. J Psychopharmacol.

[5] Greenhill LL, Posner K, Vaughan BS, Kratochvil CJ (2008). Attention deficit hyperactivity disorder in preschool children. Child Adolesc Psychiatr Clin N Am.

[6] Spira EG, Fischel JE (2005). The impact of preschool inattention, hyperactivity, and impulsivity on social and academic development: a review. J Child Psychol Psychiatry.

[7] Gresham FM, MacMillan DL, Bocian KM, Ward SL, Forness SR (1998). Comorbidity of hyperactivity-impulsivity-inattention and conduct problems: risk factors in social, affective, and academic domains. J Abnorm Child Psychol.

[8] Harpin VA (2005). The effect of ADHD on the life of an individual, their family, and community from preschool to adult life. Arch Dis Child.

[9] Barkley RA (2006). Attention deficit hyperactivity disorder: a handbook for diagnosis and treatment. 3.

[10] Ingersoll B, Goldstein S (1993). Attention deficit disorder and learning disabilities: realities, myths, and controversial treatments.

[11] Jensen PS, Garcia JA, Glied S, Crowe M, Foster M, Schlander M, Hinshaw S, Vitiello B, Arnold LE, Elliott G, Hechtman L, Newcorn JH, Pelham WE, Swanson J, Wells K (2005). Cost-effectiveness of ADHD treatments: findings from the multimodal treatment study of children with ADHD. Am J Psychiatry.

[12] van der Oord S, Prins PJM, Oosterlaan J (2012). The adolescent outcome of children with attention deficit hyperactivity disorder treated with methylphenidate or methylphenidate combined with multimodal behaviour therapy: results of a naturalistic follow-up study. Clin Psychol Psychother.

[13] Satterfield JH, Faller KJ, Crinella FM et al. A 30-year prospective follow-up study of hyperactive boys with conduct problems: adult criminality. J Am Acad Child Adolesc Psychiatry 2007; 46: 5: 601–610. 10.1097/chi.0b013e318033ff59 17450051

[14] Schachar RJ, Tannock R, Cunningham C (1997). Behavioral, situational and temporal effects of treatment of ADHD with methylphenidate. J Am Acad Child Adolesc Psychiatry.

[15] Hechtman L (2006). Long-term treatment of children and adolescents with attention-deficit/hyperactivity disorder (ADHD). Curr Psychiatry Rep.

[16] Safren SA, Otto MW, Sprich S (2005). Cognitive-behavioral therapy for ADHD in medication-treated adults with continued symptoms. Behav Res Ther.

[17] Swanson JM, McBurnett K, Christian DL (1995). Stimulant medication and treatment of children with ADHD. Adv Clin Child Psychol.

[18] Goldman LS, Genel M, Bezman RJ (1998). Diagnosis and treatment of attention-deficit/hyperactivity disorder in children and adolescents. JAMA.

[19] Storebø OJ, Ramstad E, Krogh HB, Nilausen TD, Skoog M, Holmskov M, Rosendal S, Groth C, Magnusson FL, Moreira-Maia CR, Gillies D, Buch Rasmussen K, Gauci D, Zwi M, Kirubakaran R, Forsbøl B, Simonsen E, Gluud C. Methylphenidate for children and adolescents with attention deficit hyperactivity disorder (ADHD). Cochrane Database of Systematic Reviews 2015, Issue 11. Art. No.: CD009885. DOI: 10.1002/14651858.CD009885.pub2. 10.1002/14651858.CD009885.pub2 PMC8763351 26599576

[20] Ghanizadeh A (2012). Atamoxetine for treating ADHD symptoms in autism: a systematic review. J Atten Disord.

[21] Stigler KA, Desmond LA, Posey DJ, Wiegland RA, McDougle CJ (2004). A naturalistic retrospective analysis of psychostimulants in pervasive developmental disorders. J Child Adolesc Psychopharmacol.

[22] Troost PW, Steenhhuis MP, Tuynman-Qua HG, Kalverdiik LJ, Buitelar JK, Minderaa RB, Hoekstra PJ (2006). Atamoxetine for ADHD symprtoms in children with pervasive developmental disorders. A pilot study. J Child Adolesc Psychopharmacol.

[23] Treweek S, Zwarenstein M (2009). Making trials matter: pragmatic and explanatory trials and the problem of applicability. Trials.

[24] Aman MG, Farmer CA, Hollway J, Arnold LE (2008). Treatment of inattention, hyperactivity and impulsiveness in ASDs. Child Adolesc Psychiatr Clin N Am.

[25] Maughan B, Rowe R, Messer J, Goodman R, Meltzer H (2004). Conduct disorder and oppositional defiant disorder in a national sample: developmental epidemiology. J Child Psychol Psychiatry.

[26] Peasgood T, Bhardwaj A, Biggs K, Brazier JE, Coghill D, Cooper CL, Daley D, De Silva C, Harpin V, Hodgkins P, Nadkarni A, Setyawan J, Sonuga-Barke EJS (2016). The impact of ADHD on the health and well-being of ADHD children and their siblings. Eur Child Adolesc Psychiatry.

[27] Gillberg C, Gillberg IC, Rasmussen P (2004). Co-existing disorders in ADHD—implications for diagnosis and intervention. Eur Child Adolesc Psychiatry.

[28] Koerting J, Smith E, Knowles MM (2013). Barriers to, and facilitators of, parenting programmes for childhood behaviour problems: a qualitative synthesis of studies of parents’ and professionals’ perceptions. Eur Child Adolesc Psychiatry.

[29] Relton C, Torgerson D, O'Cathain A, Nicholl J (2010). Rethinking pragmatic RCTs: introducing the ‘cohort multiple RCT’ design. BMJ.

[30] Hall SE, Riccio CA (2012). Complementary and alternative treatment use for autism spectrum disorders. Complement Ther Clin Pract.

[31] Bussing R, Zima BT, Gary FA, Garvan CW (2002). Use of complementary and alternative medicine for symptoms of attention-deficit hyperactivity disorder. Psychiatr Serv.

[32] Concannon P, Tang Y (2005). Management of attention deficit hyperactivity disorder: a parental perspective. J Paediatr Child Health.

[33] Kemper KJ, Vohra S, Walls R (2008). The use of complementary and alternative medicine in pediatrics. Pediatrics.

[34] Davis R, Mohr C (2004). The assessment and treatment of behavioural problems. Aust Fam Physician.

[35] Sanders H, Davis MF, Duncan B (2003). Use of complementary and alternative medical therapies among children with special health care needs in southern Arizona. Pediatrics.

[36] Huang A, Seshadri K, Matthews T, Ostfeld BM (2013). Parental perspectives on use, benefits, and physician knowledge of complementary and alternative medicine in children with autistic disorder and attention-deficit/hyperactivity disorder. J Altern Complement Med.

[37] Sinha D, Efron D (2005). Complementary and alternative medicine use in children with attention deficit hyperactivity disorder. J Paediatr Child Health.

[38] Strauss LC. The efficacy of a homeopathic preparation in the management of Attention Deficit Hyperactivity Disorder. Biochemical Therapy. 2000;18(2):197–201.

[39] Razlog R, Pellow J, White SJ. A pilot study on the efficacy of *Valeriana officinalis* mother tincture and *Valeriana officinalis* 3X in the treatment of attention deficit. Health SA Gesondheid. 2012; http://www.hsag.co.za/index.php/HSAG/article/view/603/779%3e. Accessed 9 Feb 2018.

[40] Lamont J (1997). Homeopathic treatment of attention deficit disorder. Br Homeopath J.

[41] Frei H, Everts R, vonAmmon K, Kaufmann F, Walther D, Hsu Schmitz S, Collenberg M, Fuhrer K, Hassink R, Steinlin M, Thurneysen A (2005). Homeopathic treatment of children with attention-deficit hyperactivity disorder: a randomised, double blind, placebo controlled crossover trial. Eur J Paediatr.

[42] Jacobs J, Williams A-L, Girard C, Yanchou Njike V, Katz D (2005). Homeopathy for attention-deficit/hyperactivity disorder: a pilot randomised-controlled trial. J Altern Complement Med.

[43] Oberai P, Gopinadhan S, Varanasi GR, Mishra A, Singh V, Nayak C (2013). Homoeopathic management of attention deficit hyperactivity disorder: a randomised placebo-controlled pilot trial. Indian J Res Homoeopathy.

[44] Barvalia P, Piyush M, Oza AH, Daftary VS, Patil VS, Agarwal ARM (2014). Effectiveness of homoeopathic therapeutics in the management of childhood autism disorder. Indian J Res Homoeopathy.

[45] Stevenson J, Buitelaar J, Cortese S (2014). The role of diet in the treatment of attention-deficit/hyperactivity disorder—an appraisal of the evidence on efficacy and recommendations on the design of future studies. J Child Psychol Psychiatry.

[46] Cooper RE, Tye C, Kuntsi J (2016). The effect of omega-3 polyunsaturated fatty acid supplementation on emotional dysregulation, oppositional behaviour and conduct problems in ADHD: a systematic review and meta-analysis. J Affect Disord.

[47] Conners, CK. (2009). Manual, Conners 3rd Edition. Multi-Health Systems Inc. New York, U.S.A.

[48] Sim J, Lewis M (2011). The size of a pilot study for a clinical trial should be calculated in relation to considerations of precision and efficiency. J Clin Epidemiol.

[49] Hayman A, Shephard N, Dimairo M, Teare MD, Walters SJ. How big should a pilot trial be? A case for continuous outcomes in randomised controlled trials. Annual Conference of the Royal Statistical Society. Telford, UK. 2012.

[50] Fibert P, Relton C, Heirs M, Bowden D (2016). A comparative consecutive case series of 20 children with a diagnosis of ADHD receiving homeopathic treatment, compared with 10 children receiving usual care. Homeopathy.

[51] Bussing R, Fernandez M, Harwood M, Hou W, Garvan CW, Eyberg SM (2008). Parent and teacher SNAP-IVratings of attention deficit hyperactivity disorder symptoms psychometric properties and normative ratings from a school district sample. Assessment.

[52] Stevens K (2012). Valuation of the Child Health Utility 9D Index. PharmacoEconomics.

[53] Becker-Witt C, Lüdtke R, Weishuhna TER, Willich SN (2004). Diagnoses and treatment in homeopathic medical practice. Forsch Komplementarmed Klass Naturheilkd.

[54] Paterson C (1996). Measuring outcomes in primary care: a patient generated measure, MYMOP, compared with the SF-36 health survey. Br Med J.

[55] National Institutes of Health/National Cancer Institute (NCI). Common Terminology Criteria for Adverse Events (CTCAE) Version 4.03. 2010. Retreived from https://evs.nci.nih.gov/ftp1/CTCAE. Accessed 9 Feb 2018.

[56] IBM Corp. Released 2013. IBM SPSS statistics for windows, version 22.0. Armonk: IBM Corp.

[57] Higgins J, Deeks J. Cochrane Handbook for Systematic Reviews of Interventions Version 5.1.0. Retrieved from The Cochrane Collaboration: 2011. www.cochrane-handbook.org.

